# Cardiotoxicity of Freon among refrigeration services workers: comparative cross-sectional study

**DOI:** 10.1186/1476-069X-8-31

**Published:** 2009-07-13

**Authors:** Laila ME Sabik, Reem A Abbas, Mahmoud M Ismail, Safwat El-Refaei

**Affiliations:** 1 Departments of Forensic Medicine and Clinical Toxicology, Faculty of Medicine-Zagazig University, Egypt; 2 Community, Environmental and Occupational Medicine, Faculty of Medicine-Zagazig University, Egypt; 3 Cardiology Department, Suez Canal Authority Hospital, Egypt

## Abstract

**Background:**

Freon includes a number of gaseous, colorless chlorofluorocarbons. Although freon is generally considered to be a fluorocarbon of relatively low toxicity; significantly detrimental effects may occur upon over exposure. The purpose of the present study is to investigate whether occupational exposure to fluorocarbons can induce arterial hypertension, myocardial ischemia, cardiac arrhythmias, elevated levels of plasma lipids and renal dysfunction.

**Methods:**

This comparative cross-sectional study was conducted at the cardiology clinic of the Suez Canal Authority Hospital (Egypt). The study included 23 apparently healthy male workers at the refrigeration services workshop who were exposed to fluorocarbons (FC 12 and FC 22) and 23 likewise apparently healthy male workers (unexposed), the control group. All the participants were interviewed using a pre-composed questionnaire and were subjected to a clinical examination and relevant laboratory investigations.

**Results:**

There were no significant statistical differences between the groups studied regarding symptoms suggesting arterial hypertension and renal affection, although a significantly higher percentage of the studied refrigeration services workers had symptoms of arrhythmias. None of the workers had symptoms suggesting coronary artery disease. Clinical examination revealed that the refrigeration services workers had a significantly higher mean pulse rate compared to the controls, though no significant statistical differences were found in arterial blood pressure measurements between the two study groups. Exercise stress testing of the workers studied revealed normal heart reaction to the increased need for oxygen, while sinus tachycardia was detected in all the participants. The results of Holter monitoring revealed significant differences within subject and group regarding the number of abnormal beats detected throughout the day of monitoring (p < 0.001). There were no significant differences detected in the average heart rate during the monitoring period within subject or group. Most laboratory investigations revealed absence of significant statistical differences for lipid profile markers, serum electrolyte levels and glomerular lesion markers between the groups except for cholesterol and urinary β2-microglobulin (tubular lesion markers) levels which were significantly elevated in freon exposed workers.

**Conclusions:**

Unprotected occupational exposure to chlorofluorocarbons can induce cardiotoxicity in the form of cardiac arrhythmias. The role of chlorofluorocarbons in inducing arterial hypertension and coronary artery diseases is unclear, although significantly elevated serum cholesterol and urinary β2-microglobulin levels raise a concern.

## Background

Fluorocarbons are a group of synthetic halogen-substituted methane and ethane derivatives containing atoms of chlorine and fluorine and are commonly known as chlorofluorocarbons (CFCs). They are generally known by commercial names such as freon, arcton and frigen [1].

Since the 1930s, when chlorofluorocarbons (CFCs), fully halogenated or non-hydrogenated fluorocarbons, were developed, they were considered to be nearly perfect chemicals. They are stable, nonflammable, noncorrosive, low in toxicity and inexpensive to produce [2]. In 1974, scientists discovered that CFCs have the capability of depleting the stratospheric ozone layer. Since the 1990s, according to the Montreal Protocol on Substances that Deplete the Ozone Layer, developed countries have replaced CFCs and hydrochlorofluorocarbons (HCFCs) (hydrogenated chlorofluorocarbons) such as dichlorodifluoromethane (CFC-12) (FC-12) (Freon 12), and chlorodifluoromethane (HCFC-22) (FC-22) (Freon 22) respectively, which cause significant stratospheric ozone depletion and global warming [3], and replaced them with hydrofluorocarbons (HFCs) (chlorine-free hydrogenated fluorocarbons), such as HFC-32 and HFC-134. However, developing countries are in the early stages of changing from CFCs to HFCs and HCFCs. The Montreal Protocol allows continued production and importation of CFCs in developing countries until 2010 to facilitate their transition to the newer CFC free technologies [4]. Although freon is generally considered to be a fluorocarbon of relatively low toxicity; significant toxic effects may occur when significant over-exposure occurs. For example, contact of the skin with the cold liquid can cause frostbite and defatting and contact with the eye may result in irritation. Inhalation of high concentrations of CFC can induce airway and respiratory problems, in addition to temporary nervous system depression with anesthetic effects. Accidental ingestion can cause nausea and ulceration of the stomach. Jaundice and mild elevations in transaminases may develop after inhalational exposure or ingestion [5-7]. Moreover, overexposure to CFCs and related halogenated hydrocarbons can cause arterial hypertension, myocardial infarction and temporary alteration of the electrical activity of the heart (atrial and ventricular cardiac arrhythmias) manifested with palpitations, irregular pulse or even inadequate circulation. Cardiac arrest and death may occur from gross overexposure [8-10].

Until now, there have been few isolated reports of cardiotoxicity and nephrotoxicity resulting from occupational exposure to different fluorocarbons. Despite the life-threatening cardiotoxic consequences of fluorinated hydrocarbon exposure, the documentation of toxic effects in human subjects is still ambiguous [1,11]. Thus, this study was conducted to determine the occupational exposure effects on the cardiovascular system and renal function when exposed to freon. We will be examining arterial blood pressure, lipid profile, heart reaction to the body's increased need for oxygen and cardiac rhythm of the cardiovascular system and the glomerular and tubular functions of the kidney based on the hypothesis that arterial hypertension in subjects with overexposure to CFCs may be precipitated by renal proximal tubular damage.

## Methods

### Study design and setting

This comparative cross-sectional study was conducted at the cardiology clinic of Suez Canal Authority Hospital in Ismailia City, Egypt.

### Subjects

Twenty three male freon-exposed workers out of 35 workers at the refrigeration services workshop (refrigeration services workers) and 23 security employees from Suez Canal Authority building (non-exposed group or the control group) who are comparable to the studied refrigeration services workers in age, sex, socioeconomic standards and duration of employment were included in this study. The participation rate was 65.7% as the ineligible participants did not fulfill the inclusion criteria mentioned below:

a) Did not agree to participate in the study; b) Were not apparently healthy; c) Had previous (prior to having been employed) occupational exposure or exposure via a secondary job to known cardio-toxins and/or nephro-toxins; and/or d) Were not free of any cardiovascular or renal disorders prior to being employed.

### Methods

#### Questionnaire

At first, the study protocol was approved by the ethics committee of Zagazig University, then after obtaining written informed consents from all the participants, they were asked to fill out a pre-composed questionnaire, which comprised of six main parts:

#### Part one

Included relevant demographic and home environment data such as age, residence and fuel used for domestic purposes.

#### Part two

Contained questions about relevant personal habits and lifestyle such as coffee consumption (>5 cups/day), salt intake (>2400 mg/day; 10 gm of salt = 2 teaspoons), consumption of fatty foods, cigarette smoking, alcohol consumption, drug use/addiction and sedentary and/or personal stress factors.

#### Part three

Contained a detailed occupational history, such as work hours, type of fluorocarbon exposure and availability of suitable protective equipment.

#### Part four

Included past medical history regarding the following:

The presence of common precipitating factors of arrhythmias such as: taking digitalis, thyrotoxicosis, pheochromocytoma, systemic infections (infective endocarditis and lobar pneumonia), myocardial damage (myocardial infarction and myocarditis) and blood loss associated with hypotension and myocardial ischemia; the presence of secondary arterial hypertension common causes such as, renal parenchymal or vascular disorder, endocrinal disorder and use of sympathomimetics or corticosteroids; the presence of precipitating factors of coronary artery disease such as, hypertension, diabetes mellitus and abnormal lipid profile.

The presence of common causes of renal impairment such as chronic systemic diseases (diabetes mellitus, hypertension, systemic lupus erythematosus, rheumatoid arthritis, etc.), infectious diseases (streptococcal infection, schistosomiasis, malaria, hepatitis B or C, HIV/AIDS, etc.), repeated urinary tract infection, obstructive uropathy and regular and prolonged intake of nephrotoxic drugs such as, antibiotics, analgesics, anti-hypertensives and anti-neoplastics.

#### Part five

Included a family history of arterial hypertension, arrhythmias or conduction disorders, nephropathy associated with hyperuricemia and gout, familial dyslipidemia and coronary artery disease.

#### Part six

Contained inquiries about symptoms suggesting:

Arterial hypertension symptoms such as dizziness, flushed face, persistent headaches, fatigue and epistaxis; coronary artery disease, such as the typical discomfort or pain felt beneath the sternum and radiating to the left shoulder, left arm, fingers, back and jaw triggered by exertion or strong emotion and subsided by rest and/or dyspnea; as well as arrhythmia, palpitations (sensation of skipped, rapid or forceful beats), dyspnea, chest discomfort, pre-syncope and syncope.

Renal involvement such as the characteristic loin pain, hematuria, polyuria, oliguria and renal edema.

### Clinical Examination

All workers were invited to attend a clinical evaluation and then subjected to the following:

-Body mass index calculation measuring the height in meters and weight in kilograms was implemented to calculate the body mass index (BMI); where the formula for BMI is weight in kilograms divided by height in meters squared [12]. This was done to exclude renal impairment, proteinuria and cardiovascular diseases that can result from obesity.

-Examination of radial artery pulse: A standardized method for radial artery pulse examination was used with an assessment of pulse rate and rhythm. Sinus rhythm was considered at the range of 60–100 beats per minute (bpm), bradycardia is considered when the rate is < 60 bpm, and tachycardia when the rate is > 100 bpm. If the time interval between beats is equal, it is considered regular and when there is a repeating irregularity, it is entered as regularly-irregular and if it is completely irregular it is called irregularly-irregular [13].

-Measurement of arterial blood pressure: Hypertension is a sustained elevation of arterial blood pressure. Repeated measurements were obtained for each subject for arterial blood pressure, which was taken three times in the early morning, using a stethoscope and a mercury sphygmomanometer. The mean of the three readings was recorded. Blood pressure is classified as normal at <120/80 mmHg, pre-hypertension at 120–139/80–89 mmHg, stage one hypertension at 140–159 mmHg (systolic) or at 90–99 mmHg (diastolic), and stage two at ≥ 160 mmHg (systolic) or ≥ 100 mmHg (diastolic) [14,15].

### Exercise Stress Testing (Exercise Electrocardiography)

Each participant in the study was subjected to exercise stress testing early in the morning, which is a screening tool to test the effect of exercise on the heart i.e. the reaction of the heart to the body's increased need for oxygen. The test gives a general sense of how healthy the heart is. A stress test is performed to determine causes of chest pain, the exercise capacity of the heart, appropriate exercise levels in those beginning an exercise program and to identify rhythm disturbances during exercise. Baseline measurements of heart rate and blood pressure were taken before the exercise started. Each participant was asked to walk on a treadmill. The pace and incline of the treadmill were gradually increased while the electrical activity of the heart was measured with an electrocardiogram (ECG) and blood pressure readings were taken. Electrodes (conductive patches) were placed on the chest, arms and legs to record the heart's activity. The blood pressure cuff on the arm was inflated every few minutes. The test continues until the participant reaches a target heart rate, unless complications such as chest pain or an exaggerated rise in blood pressure develop. Each participant was monitored for 10–15 minutes after exercising or until the heart rate returns to baseline [16].

### Ambulatory Electrocardiography (Holter Monitoring)

The freon-exposed workers (in exposed and control days) and non-exposed workers were outfitted with a holter cardiographic monitor (General Electric/SEER light ambulatory recorder/controller) that they placed on a belt around the waist. Seven adhesive electrode leads were attached to the skin of the chest. Monitoring was conducted throughout 24-hour period while the workers keep a diary of their activities. The portable recorder uses two channels to record the electrocardiogram for 24 hours. The two channels are used to record the electrocardiogram and ensures no false interpretation due to an arrhythmia. This technique is extremely useful in documenting transitory arrhythmias as supraventricular arrhythmias like (premature atrial contractions, paroxysmal atrial tachycardia, atriventricular (AV) junctional rhythms and atrial fibrillation) or ventricular arrhythmias like premature ventricular contractions and ventricular tachycardia. Interpretation and extraction of heart rate variability (HRV) parameters were performed automatically by using the software provided by General Electric (MARS PC Ambulatory ECG Analysis System), and then reviewed by a cardiologist to determine if any atypical electrical patterns. A report of the heart's activity is tabulated and irregular heart activity is correlated with the worker's activity at the time [17].

### Lipid Profile Study

Fasting lipid profile was determined through serum samples obtained from all the participants using Dimension Expanded Auto-analyzer where all the subjects fasted for a period of 12 to 14 hours before the test. Respectively, the reference intervals considered to be normal used by the laboratory of Suez Canal Authority Hospital for serum cholesterol, low density lipoprotein (LDL), high density lipoprotein (HDL) and triglycerides were, levels lower than 200 mg/dL, 60–160 mg/dL, 35–60 mg/dL and 30–200 mg/dL. The conversion factor for serum cholesterol, LDL and HDL in mg/dL to mmol/L is 0.02586 and for serum triglycerides in mg/dL to mmol/L is 0.01129.

### Assessment of Serum Electrolytes

Serum electrolytes were assessed to exclude their roles in the occurrence of arrhythmias [14,18]. The serum samples were analyzed using Dimension Expanded Auto-analyzer to determine serum sodium (Na+), potassium (k+) and calcium (Ca++) levels. The respective reference intervals used by the laboratory of Suez Canal Authority Hospital for serum Na+, K+ and Ca++ were 136–145 mmol/L, 3.5–5.1 mmol/L and 8.5–10.1 mg/dL. The conversion factor for serum Ca++ in mg/dL to mmol/L is 0.2500.

### Specific Markers for Early Renal Dysfunction

Each participant was asked to collect morning midstream urine sample in a closed sterile container. The urine samples were examined macroscopically for color and clarity, then for indicators of early renal dysfunction as follows:

assessment of microalbuminuria using Micral-Test11 strips, produced by Roche Diagnostics Company. Early effects on the glomeruli were detected by the presence of albumin in urine as it appears before clinical proteinuria. Normal urine contains albumin in a concentration of less than 20 mg/L. Dipsticks, however, only detect albumin in a concentration around 300 mg/L. An increase in albumin between these two levels is called microalbuminuria [19].

The assessment of urinary β_2_-microglobulin using ORG 5BM test, produced by ORGENTEC Diagnostika GmbH is a sensitive indicator for identifying functional and morphological changes at the proximal tubule level. Normal values for urine samples are in the range of 0–0.3 μg/mL [20].

### Environmental Assessment

To assess the occupational exposure to freon 12 and 22, we relied on NIOSH standardized sampling and analytical methods. A total of five air samples from the breathing zone of the studied refrigeration services workers were collected during the recharging of different devices. Personal sampling was conducted for freon 12 and 22 using two charcoal tubes in a series as first tube 400/200 mg, second tube 100/50 mg sections and both 20/40 mesh, which were manufactured by Sigma-Aldrich/Supelco. At the end of work shift, charcoal adsorption tubes were washed with isopropyl alcohol and the wash was then injected into gas chromatography/mass spectrometer (GC/MS) for analysis [18]. Then the averages of the five readings for freon 12 and 22 were calculated and compared to the threshold limit values of the American Conference of Governmental Industrial Hygienists (ACGIH) (TLV-TWA) (threshold limit value – time-weighted average).

### Statistical Analysis

The collected data was statistically analyzed using Epi-Info software version 6.1 [21]. Comparison between group means was done using student's t-test; while a paired t test was used for paired quantitative data and Chi square test for qualitative data. The significance level was considered at P-value < 0.05.

## Results

Table 1 demonstrates that there were no significant statistical differences between refrigeration services workers and the control group in regards to age, excess salt intake, cigarette smoking, stressful lifestyle and duration of employment, i.e. p > 0.05. Also, it was found that all of the workers in the study live in urban areas, use natural gas and electricity for domestic purposes and live a sedentary life. However, none of them consume excess coffee, fatty foods and alcohol, are not drug addicts or use personal protective measures during work. Regarding past medical and family history, there were no significant statistical differences between the two study groups as regards to the past medical history of nephrotoxic drug abuse and hepatitis C, as well as a family history of dyslipidemia and/or hypertension and coronary artery disease. However, none of the workers in the study had a past medical history of precipitating factors indicating arrhythmias, coronary artery disease, secondary arterial hypertension causes or had a family history of arrhythmias or nephropathy associated with hyperuricemia and gout.

**Table 1:** Relevant demographic characteristics, occupational data and relevant past medical and family history of the studied workers by Chi Square test:

Relevant characteristics and occupational data	Freon-exposed workers (N = 23)	Non-exposed workers (N = 23)	P-value
Age (years)( ± SD)	45.9 + 4.4	43.7 ± 3.2	0.058

Special habits & life style N (%)			
Excess salt intake	14 (60.9%)	9 (39.1%)	0.14
Cigarette smokers	11 (47.8%)	7 (30.4%)	0.22
Stressful life	14 (60.9%)	17 (73.9%)	0.34

Duration of employment (years) ( ± SD)	20.9 + 3.3	19.3 ± 2.7	0.078

**Relevant past medical and family history**			

Past history of nephrotoxic drug abuse N (%)	6 (26.1%)	3 (13.04%)	0.45
Past history of hepatitis C N (%)	2 (8.7%)	4 (17.4%)	0.66

Family history of dyslipidemia and/or hypertension N (%)	19 (82.6%)	13 (56.5%)	0.054
Family history of coronary artery disease N%)	4 (17.4%)	1 (4.3%)	0.34

**Figure 1:**
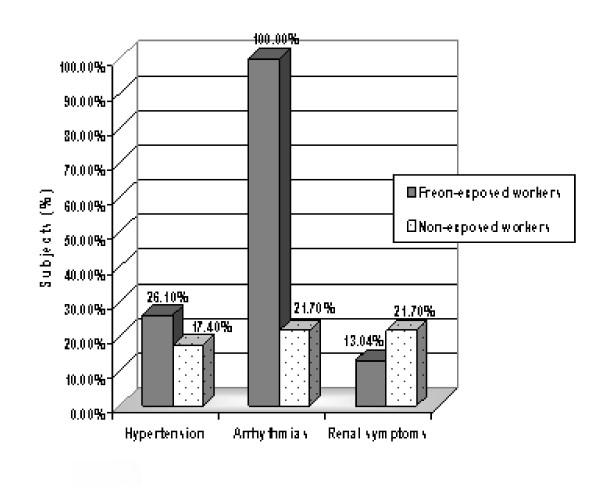
**A percent distribution of the studied workers regarding present symptoms suggesting hypertension, arrhythmias, and renal disease**.

Figure 1 demonstrates that there were no significant statistical differences between the two study groups in regards to symptoms suggesting hypertension and renal disease, p > 0.05. While a significantly higher percent of the exposed workers had symptoms suggesting they have arrhythmias compared to the control group, p < 0.001. However, none of the studied workers had symptoms indicating coronary artery disease.

Table 2 illustrates that there were no significant statistical differences between the two study groups in regard to body mass index and arterial blood pressure (systolic and diastolic)(p > 0.05). However, the mean pulse rate was significantly elevated in the exposed workers compared to the control group.

**Table 2:** Clinical examination and laboratory investigations results of the studied workers by student's t-test:

Clinical examination results	Freon-exposed workers (N = 23)	Non-exposed workers (N = 23)	P-value
BMI (Kg/m^2^) ( ± SD)	29.9 ± 2.3	30.2 ± 3.3	0.72

Pulse rate (bpm) ( ± SD)	97.8 ± 11.9	81.3 ± 3.9	0.018

Arterial blood pressure (mmHg) ( ± SD)			
Systolic blood pressure	130.0 ± 5.9	128.0 ± 4.3	0.19
Diastolic blood pressure	81.8 ± 5.2	80.3 ± 3.4	0.25

**Laboratory investigations results**			

Lipid profile (mmol/L) ( ± SD)			
Cholesterol	5.33 ± 23.6	4.71 ± 15.7	0.0002
LDL	3.57 ± 25.5	3.75 ± 17.2	0.29
HDL	0.93 ± 7.4	0.96 ± 3.9	0.41
Triglycerides	2.15 ± 52.2	2.02 ± 31.5	0.34

Serum electrolytes (mmol/L) ( ± SD)			
Na+	140.9 ± 2.7	141.7 ± 3.3	0.31
K+	4.4 ± 0.2	4.7 ± 0.7	0.17
Ca++	2.23 ± 0.4	2.15 ± 0.8	0.21

Renal dysfunction markers ( ± SD)			
Urinary microalbumin (mg/dl)	1.7 ± 1.5	18.3 ± 3.3	0.19
Urinary β_2_-microglobulin (μg/ml)	0.51 ± 0.1	0.22 ± 0.04	0.0012

Regarding laboratory investigations, there were no significant statistical differences between the two study groups regarding lipid profile markers, serum electrolytes levels, and glomerular lesion marker (urinary microalbumin). However, total cholesterol level and urinary β_2_-microglobulin (tubular lesion marker) were significantly elevated in the exposed workers compared to the control group.

In the present study, exercise stress testing for the studied workers revealed normal heart reactions to the body's increased need for oxygen; where sinus tachycardia was detected in all the participants.

**Figure 2:**
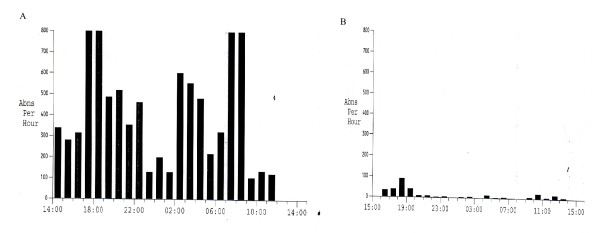
**(a) Narrative summary of Holter monitoring for Freon-exposed worker during an exposed day showed frequent atrio-ventricular multifocal arrhythmias**. **(b) **Narrative summary of Holter monitoring for the same Freon-exposed worker during a control day showed a lower frequency of abnormal beats.

**Figure 3:**
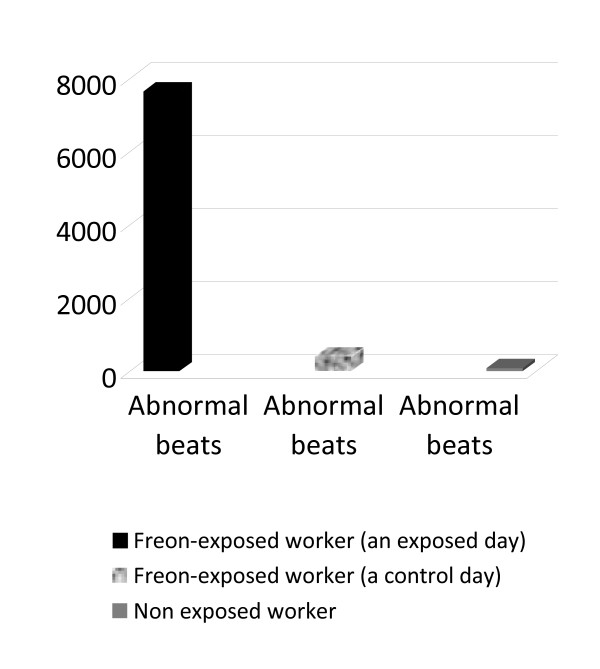
**A mean of studied workers regarding abnormal beats**.

**Figure 4:**
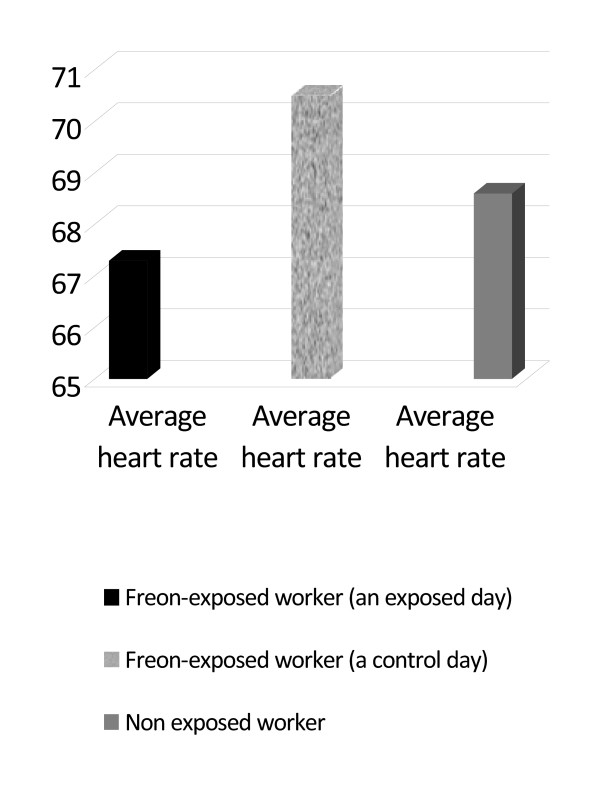
**A mean of studied workers regarding average heart rate**.

As to the Holter monitoring results, the recording was reviewed for any atypical electrical patterns. Premature atrial and ventricular multifocal arrhythmias were the only detected arrhythmias in freon-exposed workers in the present study. Holter monitoring results for freon-exposed workers on exposed workdays and on another control days revealed significant statistical differences within subjects as regards to the number of abnormal beats (with a mean of 7645.7 ± 1999.7 and 405.9 ± 104.2 respectively), which were detected throughout the day of monitoring (p < 0.001). Also, freon-exposed workers experienced a statistically significant higher mean of abnormal beats compared to the control group (with a mean of 7645.7 ± 1999.7 and 84.9 ± 16.2 respectively)(p < 0.001) (Figures 2&3). In regard to the average heart rate during the monitoring period, no significant differences within subjects were detected on exposed workdays and on other control days (with a mean of 67.3 ± 12.3 and 70.5 ± 2.4 respectively) or within study groups (with a mean of 67.3 ± 12.3 and 68.6 ± 17.5 respectively) (Figure 4).

Table 3 illustrates that the average concentrations of freon 12 and 22 in the breathing zone of refrigeration workers were 5720 mg/m^3 ^and 4310 mg/m^3 ^respectively, which exceed threshold limit values, which are 4950 mg/m^3 ^and 3540 mg/m^3 ^respectively.

**Table 3:** Average concentration of freon 12 and 22 in workshop of refrigeration workers:

Average concentration of freon 12 and 22 in the refrigeration services workshop	The corresponding Threshold limit value (TLV)mg/m^3^
Freon 12 5720 mg/m^3 ^± 580	4950 mg/m^3^

Freon 22 4310 mg/m^3 ^± 420	3540 mg/m^3^

## Discussion

CFCs have been used as refrigerants, solvents, foam-blowing agents, aerosol propellants, fire-extinguishing agents, anesthetics, insecticides, fungicide and as intermediates for the production of polymers [2]. Due to the anticipated widespread use of CFC replacements, there has been a major effort to characterize their direct toxic effects on humans and their effects on the environment [22].

In the present study, there were no significant statistical differences between refrigeration services workers and the control group in regards to symptoms suggesting arterial hypertension and measurements of arterial hypertension. However the two study groups were in the pre-hypertension stage. Also, none of the studied workers had symptoms suggesting coronary artery disease. Moreover, exercise stress testing for the studied workers revealed normal heart reaction to the increased need for oxygen, where sinus tachycardia was detected in all the participants. This could suggest that freon-exposed workers under normal working conditions are not at an increased risk for arterial hypertension or coronary artery disease.

However, two cases of acute massive freon exposure preceded secondary arterial hypertension and a fatal case of myocardial infarction occurred in an elderly man exposed to decomposed freon-22 [9,23]. Moreover, clinical pathologists exposed to fluorocarbons during the preparation of frozen tissue sections have been found to develop coronary heart disease to an increased degree [24]. This discrepancy of the results between the present study and the other studies could be attributed to exposure of workers to different freon levels and types as well as to individual susceptibility, where individuals with preexisting conditions of the cardiovascular system and/or concurrent stress (associated with elevated levels of catecholamines) may have increased susceptibility to the toxicity of excessive freon exposure [5,25].

The present study revealed that there were no significant statistical differences between the two study groups in regards to lipid profile markers, except total cholesterol level, which was significantly elevated in the exposed workers compared to the control group. Our findings partially agree with previous study results in which serum cholesterol levels were slightly higher and triglyceride levels were lower in adult male rats exposed to 50, 000 ppm freon 22 for five hours per day for eight weeks [26].

In the present study, there were no significant statistical differences between the groups studied regarding causes and risk factors for abnormal lipid profile, such as eating fatty foods, sedentary lifestyle, obesity, stress and familial dyslipidemia [27]. Thus, it is possible that occupational exposure to freon may be responsible for an abnormal increase in total cholesterol level, which in turn might increase the risk of cardiovascular disorders, especially coronary artery disease, among freon-exposed workers.

In this study, a significantly higher percent of the refrigeration services workers complained of symptoms suggesting arrhythmias, as compared to the non-exposed workers. Also, the mean pulse rate was significantly elevated in the exposed group compared to the control group, although it was still within the normal range in the two study groups. Furthermore, the results of Holter monitoring revealed statistically significant differences within subjects as regards the number of abnormal beats detected throughout the day of monitoring (multifocal AV arrhythmias). Also, freon-exposed workers experienced a statistically significant, higher mean of abnormal beats detected throughout the day of monitoring compared to the control group.

In the present study, there were no significant statistical differences between the two study groups in regards to some of the precipitating factors for arrhythmias, such as age and serum levels of electrolytes, which were within the normal range for the two study groups. Also, none of the workers in the study group had past medical histories suggesting the presence of any precipitating factors for arrhythmia or occupational exposure to arrhythmogenic chemicals, or had family history of arrhythmias [17,28]. So, it could be concluded that freon-exposed workers are at increased risk for cardiac arrhythmias.

Contrary to our findings, many epidemiologic studies of fluorocarbons exposure have not found a significant increase in arrhythmia frequencies under normal working conditions. However, these studies don't negate the potential toxicity of these substances in situations where exposures are high, such as in a confined space, after a spill or in individuals with underlying heart disease [28]. In a study in which ambulatory (ECG) were recorded for 24 hours on the day of exposure, and on a control day for refrigerator repairmen exposed to FC 12 and FC 22, and on any work day for a control group, no clear connection between fluorocarbons and cardiac arrhythmia was found, although one subject had several ventricular ectopic beats, which may have been with the result of exposure [29]. Also, in a field study conducted on refrigerator repairmen exposed to FC 12 and FC 22, two types of arrhythmia were recorded, ectopic beats and sudden bradycardia. However, no significant differences between exposed and unexposed periods, and no consistent dose effect relations were observed. Thus, the results did not support the notion that fluorocarbons induce cardiac arrhythmia in occupationally exposed refrigerator repairmen [30]. Moreover, the absence of subject differences in the rate of ventricular and supraventricular arrhythmias were recorded among healthy workers cleaning rocket and ground support equipment for the National Aeronautic and Space Administration (NASA) programs and exposed to fluorocarbon 113. This result was attributed to low level of FC113 exposures, which was below (OSHA) 8-hour (TWA) (PEL) and (NIOSH) recommended exposure limit (REL) of 1000 parts per million (ppm), that did not induce cardiac arrhythmias or subtle changes in cardiac activity [31]. This is a very important result, as despite that FC 113 has a high level of toxicity, keeping workers' exposure below the recommended levels can prevent its toxic effects [32].

However the results of many historical studies agree with that of the present study. In one study, all pathology residents in a Boston hospital experienced palpitations that appeared to be associated with the addition of the surgical pathology rotation to their schedules. On this rotation, the only procedure that could have possibly caused palpitations was the preparation of frozen sections in which a freon-22-based aerosol was used to decrease work time [33]. Also, a number of deaths have been reported among persons "sniffing" freon accidentally and intentionally [33,34]. In addition, a case of ventricular fibrillation and two cases of atrial fibrillation were recorded after exposure to a fluorinated hydrocarbon and (CFC 113) respectively [35,36].

There are several hypotheses on the mechanism for the arrhythmogenic effect of hydrocarbons and fluorocarbons including: the effects on the central nervous system, respiratory reflexes, changes in potassium levels, or either the adrenergic and/or cholinergic receptors; the direct effects on the cardiac conduction system; or the sensitizing of the heart to the endogenous catecholamines [28,37]. Another theory suggests that CFCs don't sensitize the myocardium to endogenous neurotransmitters, but have a depressant effect on the sinus atrio-ventricular and ventricular conduction systems, allowing other ectopic foci to produce arrhythmias [1]. Moreover, it was found that, fluorocarbons produce both hypertension and coronary artery disease through damage to the endothelial barrier in the vascular system, activation of leukocytes and platelets, initiation of plaque formation, stimulation of the inflammatory response, kidney-related hypertension, and direct damage to cardiac and blood vessel tissue [38].

Individuals with preexisting diseases/conditions of the cardiovascular system or under circumstances where an abnormally large amount of adrenaline is secreted endogenously (such as anger, fear, excitement and severe exertion) or exposed to epinephrine or other sympathomimetic amines e.g. bronchodilator and nasal decongestant, may have increased susceptibility to the toxicity of excessive exposure [5,25]. Furthermore, the arrhythmogenicity of freon on the heart conduction system is enhanced synergistically by hypoxia [39]. Also, caffeine intake, physical activity and tobacco use have been suggested to have a pro-arrhythmic effect [1].

The results of the present study revealed an absence of a statistically significant difference between the two study groups regarding glomerular lesion markers (urinary microalbumin), while the level of urinary β_2_-microglobulin (tubular lesion marker) was significantly elevated in freon-exposed workers compared to the control group. In the present study, there were no statistically significant differences between the two study groups regarding age, residence, type of domestic fuels, excess salt intake, obesity, cigarette smoking, alcohol consumption, past history of chronic systemic diseases, certain infectious diseases, complicated urinary tract infection, obstructive uropathy and excess intake of nephrotoxic drugs. Additionally, none of the workers had previous jobs and/or secondary occupations exposing them to different nephrotoxins, nor had family history of nephropathy associated with hyperuricemia and gout [40,41]. So, we suggested that exposure to freon may be an important precipitating factor for renal tubular damage and subsequently renal impairment, which is an important cause for secondary arterial hypertension [14]. These results agree with Brogren et al [42], who reported that organic chlorinated hydrocarbon compounds are known to be nephrotoxic through induction of an autoimmune necrosis of renal tubular cells. In addition, it was hypothesized that freon-induced-arterial hypertension was precipitated by renal proximal tubular damage [8,9].

The average airborne concentrations of freon 12 and 22 at the refrigeration workshop during recharging and repairing activities in the present work were found to exceed the corresponding ACGIH TLV (TWA). These results disagree with the findings of other researchers, who revealed that exposure of volunteers to CFC-11 at concentrations of 250, 500, or 1000 ppm for periods of 1 min to 8 hr, did not produce any untoward physiological effects. Exposure for 8 hr/day, 5 days/week for 2 to 4 weeks to CFC-11 at a concentration of 1000 ppm showed only minor decrement in several cognitive tests [31]. There is also a disagreement regarding the findings of WHO [43], where workers with refrigerant equipment with mean exposure time was 10 min., and chlorofluorocarbon concentrations in their breathing zone exceeded 750 ppm. Their cardiac arrhythmias were registered before, during, and after the exposure by means of a portable ECG. No statistically significant difference was found between exposed and non-exposed periods, nor was there any dose related trend for different individuals when grouped into different exposure groups. The conflict between these studies and the present study findings may not only be due to low concentration levels, but also due to short exposure times during these studies. Thus from our results, it could be concluded that the detected cardiovascular changes besides elevated serum cholesterol and urinary β_2_-microglobulin (tubular lesion marker) levels among the refrigeration services workers could be attributed to freon exposure exceeding the threshold limit value resulting from leakage during the recharging and repairing of the devices.

## Conclusions

In conclusion, the data indicate that unprotected occupational exposure to freon in the present workplace can induce cardiotoxicity, mainly in the form of cardiac arrhythmias, though their role in inducing arterial hypertension and coronary artery diseases is not well established despite significantly elevated serum cholesterol and urinary β_2_-microglobulin (tubular lesion marker) levels. Moreover, the nephrotoxicity of freon in the studied workers was detected at the level of the tubules, while no glomerular affection was detected. Finally, environmental assessment results for freon in the refrigeration services workshop were found to exceed the threshold limit value. Therefore, substitution of the non-hydrogenated fluorocarbons (CFC) and chlorine-containing hydrogenated fluorocarbons with chlorine-free hydrogenated fluorocarbons, after toxicity testing of these substitutes on both animals and humans, in addition to improved working conditions, are strongly recommended.

## Abbreviations

A.C.G.I.H: (American Conference of Governmental Industrial Hygienists); AV: (atrioventricular); BMI: (body mass index); bpm: (beats per minute); CFC: (chlorofluorocarbons); CNS: (central nervous system); ECG: (electrocardiogram); FC 12: (freon 12), (fluorocarbon 12); FC 22: (freon 22), (fluorocarbon 22); GC-MS: (gas chromatography/mass spectrometer); HDL: (high density lipoprotein); HFC: (hydrofluorocarbons); HRV: (heart rate variability); LDL: (low density lipoprotein); NASA: (National Aeronautic and Space Administration); NIOSH: (National Institute for Occupational Safety and Health); OSHA: (Occupational Safety and Health Administration); PEL: (permissible exposure limit); ppm: (parts per million); REL: (recommended exposure limit); TLV: (threshold limit value); TWA: (time-weighted average); WHO: (World Health Organization).

## Competing interests

The authors declare that they have no competing interests.

## Authors' contributions

LMES designed the study, data collection and analysis, wrote and reviewed the manuscript. RAA chose the studied groups and analyzed their working conditions, conducted environmental assessments, performed the statistical analysis, participated in the data analysis, participated in, wrote and reviewed the manuscript. MMI was involved in revising the manuscript; SER conducted the research and participated in data analysis. All authors read and approved the final manuscript.
